# Influence of Strain Rate on Barkhausen Noise in Trip Steel

**DOI:** 10.3390/ma17215330

**Published:** 2024-10-31

**Authors:** Martin Pitoňák, Anna Mičietová, Ján Moravec, Jiří Čapek, Miroslav Neslušan, Nikolaj Ganev

**Affiliations:** 1 Faculty of Civil Engineering, University of Žilina, Univerzitná 1, 010 26 Žilina, Slovakia; martin.pitonak@uniza.sk; 2 Faculty of Mechanical Engineering, University of Žilina, Univerzitná 1, 010 26 Žilina, Slovakia; anna.micietova@fstroj.uniza.sk (A.M.); jan.moravec@fstroj.uniza.sk (J.M.); 3 Faculty of Nuclear Sciences and Physical Engineering, Czech Technical University in Prague, Trojanova 13, 120 00 Prague, Czech Republic; jiri.capek@fjfi.cvut.cz (J.Č.); nikolaj.ganev@fjfi.cvut.cz (N.G.)

**Keywords:** trip steel, Barkhausen noise, tensile test, strain rate, plastic straining, self-heating

## Abstract

This paper deals with Barkhausen noise in Trip steel RAK 40/70+Z1000MBO subjected to uniaxial plastic straining under variable strain rates. Barkhausen noise is investigated especially with respect to microstructure alterations expressed in terms of phase composition and dislocation density. The effects of sample heating and the corresponding Taylor–Quinney coefficient are considered as well. Barkhausen noise of the tensile test is measured in situ as well as after unloading of the samples. In this way, the contribution of external and residual stresses on Barkhausen noise can be distinguished in the direction of tensile loading, as well as in the transversal direction. It was found that the in situ-measured Barkhausen noise grows in both directions as a result of tensile stresses and the realignment of domain walls. The post situ-measured Barkhausen noise drops down in the direction of tensile load due to the high opposition of dislocation density at the expense of the growing transversal direction due to the prevailing effect of the realignment of domain walls. The temperature of the sample remarkably grows along with the increasing strain rate which corresponds with the increasing Taylor–Quinney coefficient. However, this effect plays only a minor role, and the density of the lattice imperfection expressed especially in terms of dislocation density prevails.

## 1. Introduction

The motion of dislocations, their interference, and their increasing density are the prevailing mechanisms of the strain hardening of ferritic steels beyond their yielding [1,2]. Prolonged plasticity (elongation at break) is usually compensated by their lower yield as well as ultimate strengths. On the other hand, the strain-induced transformation of austenite to martensite can be found as the predominating mechanism of strain hardening for some austenitic steels [3,4,5]. This phenomenon is well known as the transformation-induced plasticity (Trip) effect [6]. This Trip effect delays the phase of strain instability as well as the corresponding elongation at break. Moreover, the phase transformation contributes to the increasing heating especially at higher strain rates when the adiabatic conditions are met [7,8]. Trip steels are considered bodies with complex microstructure containing a mixture of ferrite, bainite, or primary martensite and austenite [4,9]. The increased strength, delayed plastic instability, and prolonged elongation at break of these bodies are due to the superimposing contribution of two main strain hardening mechanisms such as dislocation slip and the strain-induced transformation of austenite to martensite [10,11,12]. Trip steels are produced in a diversity of microstructures in which the chemistry and/or fraction of the aforementioned phases can vary in order to customise the final mechanical properties and the corresponding formability. These materials are very often employed in the automotive industry for body-in-white parts requiring high crashworthiness, reinforced components, bumpers, etc. [13]. Moreover, these steels are employed in civil engineering as well for components that transform the energy of impacts into the energy consumed during their plastic deformation.

Alterations in microstructure during the plastic deformation of Trip steels are very complex. The mechanism of dislocations is directly stored in the matrix with respect to its increasing density, whereas the valuable sample heating can be obtained at higher strain rates mostly [14]. However, the strain-induced transformation of austenite to martensite contributes to the more intensive sample heating as well [8,15]. Moreover, the rate of austenite decomposition decelerates along with the increasing strain hardening in the Trip steels containing lower C content [16,17,18,19].

An increasing degree of matrix alteration expressed in terms of an increasing density of lattice imperfection can be analysed using the Taylor–Quinney coefficient (*β_INT_*) [12,13]. The values of this coefficient near zero indicate that nearly whole plastic work is stored in the matrix in the form of lattice imperfections, whereas a coefficient near 1 indicates a nearly unaffected matrix and the transformation of plastic work into sample heating. The Taylor–Quinney coefficient can be obtained from the first law of thermodynamics when the adiabatic conditions are met [14,20]:

(1) $$\beta_{INT}=\frac{\rho C_{p}∆T}{\int dW_{p}}$$

where *W_p_* is the incremental plastic work, *ρ* is the matrix density, *C_p_* refers to the heat capacity, and *∆T* is the temperature growth. *β_INT_* can exceed 1 when another source such as phase transformation is involved [8]. Rittel et al. [20] investigated *β _INT_* in Ti, Al, and Fe alloys and found that *β _INT_* is higher in materials with poor thermal conductivity. Zaera et al. [8] found that *β _INT_* is higher for austenitic steels due to the Trip effect. Smith [21] studied the Taylor–Quinney coefficient as a function of strain rate. Soares et al. [14] and Rittel [22] also studied the influence of strain rate on *β_INT_* in high-entropy alloys and glassy polymers.

Magnetic Barkhausen noise (MBN) is affected by lattice imperfections due to their encountering by domain walls (DWs) in motion [23,24]. The origin of MBN is directly connected to the presence of pinning sites blocking DWs in their positions and their sudden irreversible and discontinuous motion as soon as the magnetic field attains the critical threshold [24,25]. The superimposing contribution of stresses should be considered, which also reorganise (realign) DWs [26,27]. The MBN technique can be used for monitoring the components in real industry (grinding, heat treatment, etc. [28,29,30]) or/and as a reliable and very fast tool for the characterisation of materials with respect to their stressing or/and microstructure alterations [31,32].

The MBN technique has already been employed for the investigation of Trip steels. Lindgren and Lepistö [33] investigated MBN anisotropy during uniaxial plastic straining. Neslušan et al. [34] reported the deceleration of austenite transformation in Trip steel beyond the yielding and the decreasing MBN after a tensile test due to the increased dislocation density. Similar conclusions were reported by Vértesy et al. [35]. Tavares et al. [36] correlated MBN with martensite/austenite partitioning in stainless steel. However, the contribution of increasing ferrite phase on MBN in Trip steels is only minor [34].

MBN has been reported as a suitable technique for monitoring Trip steels, especially with respect to their microstructure. However, the degree of these alterations can be affected by strain rates as well as the developed strains [14,21] due to altering the ratio between the plastic work consumed for heating and the increasing density of lattice imperfection. As mentioned above, this behaviour is quite complex in Trip. Furthermore, Trip steels are usually employed for components consuming the energy under random impacts when high strain rates are developed and kept within the whole deformation process (for example, under traffic collisions). The evolution of component deformation, microstructure alterations, phase stability, and their residual stress state is driven by the variety of mechanisms whose contribution is mixed and difficult to unwrap. Strain rates very often play a significant role in the aforementioned behaviour and might therefore affect Barkhausen noise emission as well. For these reasons, this study investigates this topic by employing the Taylor–Quinney coefficient. Furthermore, this study investigates the influence of the variable strain rate on Barkhausen noise emission when the contribution of the residual stresses (post situ measurements of MBN), the external stressing (in situ measurements of the tensile test), and the microstructure alterations are investigated as well.

## 2. Materials and Methods

### 2.1. Materials 

**Figure 1:**
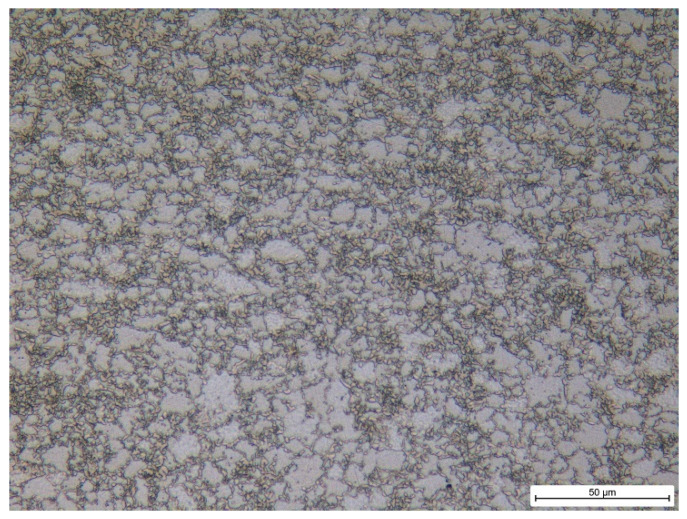
Metallographic image of the investigated Trip steel, 3%Nital.

**Figure 2:**
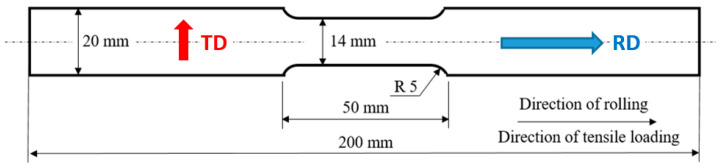
Gauged sample for the uniaxial test.

Experiments were carried out on galvanised Trip steel RAK 40/70+Z1000MBO. The microstructure of the investigated steel (as-received) is illustrated in Figure 1. The as-received microstructure of this Trip steel is composed of austenite (13.6%), ferrite (47.6%), bainitic ferrite (28.3%), and martensite (10.5%) [34]. The way in which the fraction of the particular phases was obtained was reported earlier [34]. The samples for the uniaxial tensile test as shown in Figure 2 were cut from a sheet of thickness 0.75 mm. Each test was repeated 3 times. The surface of the sheet was subjected to galvanising and the thickness of the Zn-galvanised layer ranged from 6 to 7 μm. The chemical composition can be found in Table 1 and the mechanical properties in Table 2.

### 2.2. MBN Measurements

MBN measurements were carried out in situ as well as after the unloading of samples in the predefined plastic strains. The in situ measurements were performed using the software ViewScan 4.0.0 CZ through the whole stress–strain curves in the frequency range of MBN from 70 to 200 kHz. The changing magnetic field was in the direction of the Trip rolling direction (RD) as well as in the transversal direction (TD) (see Figure 2). In order the obtain a richer MBN signal (MBN signal in the frequency range from 10 to 1000 kHz), MBN was also measured in situ using software MicroScan 5.4.1 in the predefined plastic strains (6%, 10%, 20%, and 27.5%) for four strain rates (1.67 × 10^−3^·s^−1^, 8.33 × 10^−3^·s^−1^, 25.00 × 10^−3^·s^−1^, and 41.67 × 10^−3^·s^−1^). The uniaxial tensile test was performed using Instron 5985 (Instron, Norwood, MA, USA) true strains were checked by the 2620-602 extensometer). Apart from the conventional effective (rms) value of the signals referred to as MBN, the MBN envelopes and the corresponding *PP* value (refers to the position of the magnetic field in which MBN attains a maximum) were also analysed as well. MBN was also measured after the unloading of samples (post situ) when the tensile test was stopped at the aforementioned plastic strains. A magnetising voltage of 5 V and magnetising frequency of 125 Hz were obtained using the voltage and frequency sweeps. The MBN signal was sampled at a frequency of 6.7 MHz. MBN measurements were carried out through the galvanised layer using sensor S1-18-12-01. The Barkhausen noise sensor (Stresstech Oy, Jyväskylä, Finland) was calibrated by the use of the Barkhausen noise reference sample Emuge 2267523/20-11 of hardness 60 HRC made of Vanadis 4 sintered powder (Stresstech Oy, Jyväskylä, Finland). Calibration was carried out before MBN measurements and verified after measurements as well. This sintered sample provides long-term and very stable MBN emission and enables stable Barkhausen noise emission of the employed sensor to be obtained.

### 2.3. Microhardness and Temperature Measurements

All measurements were carried in the centre of the gauged part with respect to its width as well as length. Microhardness (*HV1*) was measured using an Innova Test 400^TM^ (INNOVATEST Europe BV, Maastricht, The Netherlands) tester (five repetitive measurements for each plastic strain and strain rate). Information about *W_p_* was calculated on the basis of the loading force exported by the Instron software. *ρ* = 7850 kg·m^−3^ and *C_p_* = 450 J·kg^−1^K^−1^ were employed for the *β_INT_* calculations. Temperature *T* was measured during the tensile test using sensor PPG101A6 (software Tera Term 5.3, sampling frequency 100 Hz).

### 2.4. XRD Measurements

Finally, X-ray diffraction (XRD) measurements were carried out as well in order to measure residual stresses, dislocation density, phase composition, and texture (crystallographically preferred orientation) after unloading using the X’Pert PRO MPD (Malvern Panalytical, Malvern, UK) diffractometer with chromium and cobalt radiation. The galvanised Zn layer was etched before the XRD measurements. Diffraction angles 2*θ* were determined using the Pearson VII function and Rachinger method from the diffraction lines Cr*Kα_1_* of the *{211}* planes of the ferrite phase. The Winholtz and Cohen method and the X-ray elastic constants ½*s*_2_ = 5.75 TPa^−1^ and *s*_1_ = −1.25 TPa^−1^ were used to determine residual stresses. Texture analysis was based on the orientation distribution function calculation from experimental pole figures (three diffraction lines *{200}*,*{211}*,*{220}*, and the MATLAB^TM^ toolbox MTEX software 5.11.2 was used for data post-processing [37]). The dislocation density was calculated on the basis of the Williamson and Smallman method [38]. It is necessary to mention that XRD is not able to directly distinguish ferrite, bainite, and martensite in low-carbon steels, because of the small tetragonality of martensite, i.e., the observed diffraction maxima overlap.

### 2.5. Experimental Plan

The experiments comprised the following:

1. In situ measurements during the tensile test such as
  (a) Stress–strain evolution;
  (b) MBN measurements in the RD and TD;
  (c) Temperature measurements.
2. Post situ measurements after the tensile test such as the following:
  (a) Non-destructive measurements such as
    - MBN measurements in the RD and TD;
    - XRD measurements in the RD and TD.
  (b) Destructive measurements such as
    - Microhardness measurements.

The time sequence of the consecutive steps was directly linked with the items listed above in the same order.

## 3. Results of Experiments and Their Discussion

### 3.1. Mechanical Properties and the Taylor–Quinney Coefficient

**Figure 3:**
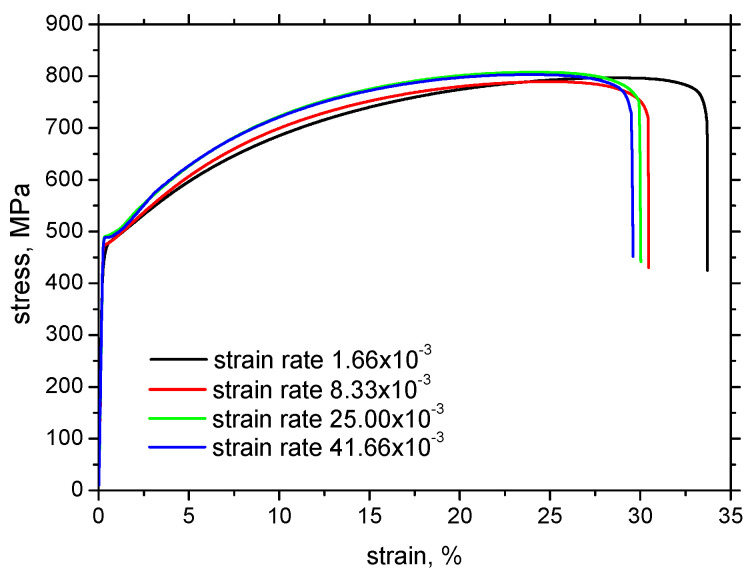
Stress–strain curves for the different strain rates.

**Figure 4:**
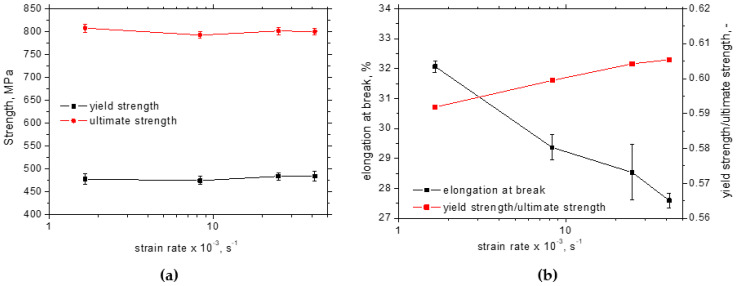
Mechanical properties as a function of strain rate: (**a**) yield and ultimate strength, (**b**) elongation at break.

**Figure 5:**
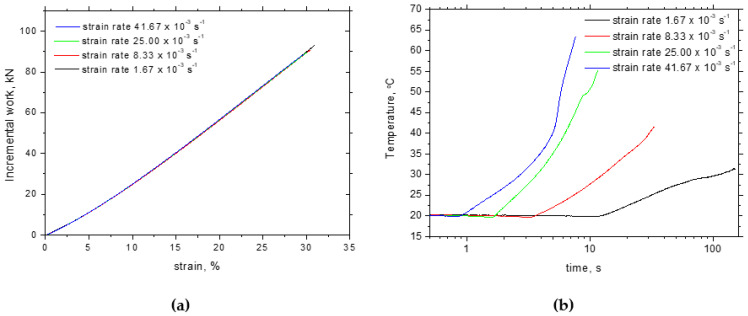
Evolution of incremental work and temperature during tensile test: (**a**) incremental work, (**b**) temperature.

Figure 3 illustrates the stress–strain curves for the different strain rates. Yield and ultimate strengths as well as the elongation at break were extracted from these curves (three repetitive measurements for each strain rate). Figure 4 demonstrates that the yield as well as the ultimate strength are nearly unaffected (together with their ratio), whereas the elongation at break gently drops down when the plastic deformation is accelerated. Also, the evolutions of incremental work plotted along with strain hardening overlap each other (see Figure 5a). On the other hand, the evolution of temperature is remarkably different at the higher strain rates as compared with the lower ones. Temperature growth becomes higher and the increase in temperature is accelerated at the higher strain rates (see Figure 5b). Two different aspects should be noted with respect to the different temperatures. The temperature is a quantity attributed to the motion of atoms in the lattice. It should be considered that the speed of dislocations is accelerated at higher strain rates. Therefore, the energy stored in the atoms when the dislocation encounters the lattice is higher. The second aspect is associated with the thermal conductivity during the tensile test. Conditions for the adiabatic process can be met at the higher strain rates mainly. However, certain heat transfers from the gauged to the neighbouring regions take place, which contributes to the lower temperatures at the lower strain rates as illustrated in Figure 5b. The increase in temperature is higher than that reported before [14] due to the contribution of an additional heat source when the strain-induced transformation of austenite to martensite as the typical behaviour of the improved formability of Trip steels takes place [11].

**Figure 6:**
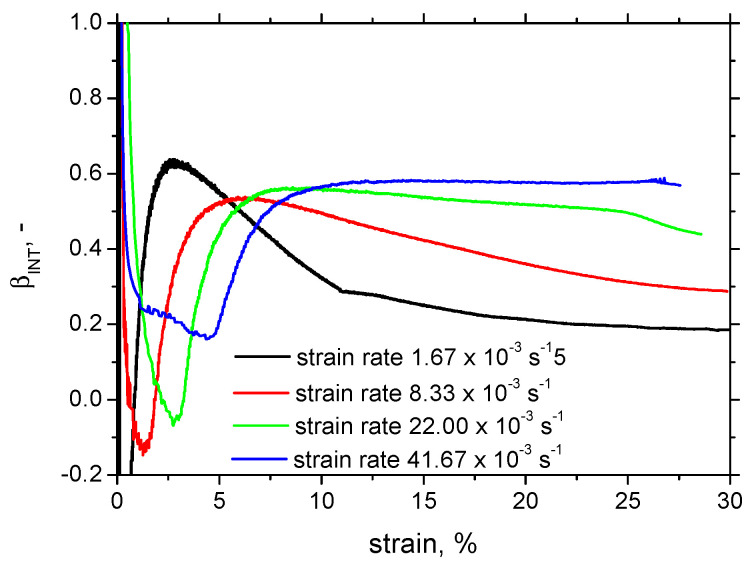
Evolution of *βINT* during tensile test.

With the remarkably different evolution of the temperature *T* for the same *W_p_*, the different evolution of *β_INT_* can be calculated and plotted as depicted in Figure 6. A more remarkable temperature growth can easily be linked with the higher *β_INT_* at the higher strain rates. *β_INT_* evolutions exhibit local minima at lower strain rates followed by local maxima at higher strains, with a final progressive decrease afterward. The local minima are lower and shifted to lower strains at the lower strain rates. The rate of the progressive decrease drops down at the higher strain rates. The lower and higher evolutions of *β_INT_* at the lower strains are due to less developed lattice alterations (early beyond the yielding), as well as due to the only-minor temperature growth. For this reason, the calculation of *β_INT_* early beyond the yielding is quite tricky. As soon as the strain hardening as well as the temperature increase are more developed, *β_INT_* stabilises and can be considered the parameter linked with the degree of the aforementioned aspects.

### 3.2. XRD and Hardness Measurements

**Figure 7:**
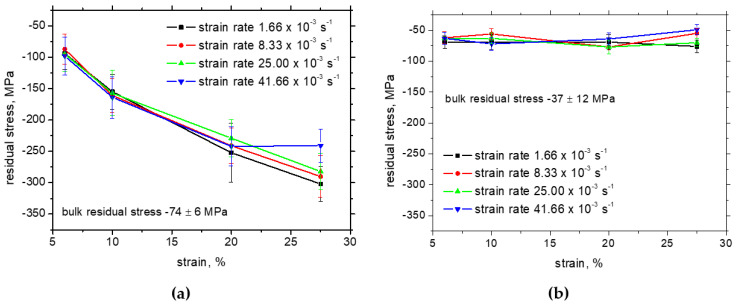
Evolution of residual stresses in ferrite along *ε*: (**a**) RD, (**b**) TD.

Figure 7b illustrates that the residual stresses in the TD are unaffected with respect to strain hardening, and the strain rate more or less corresponds to the bulk compressive stresses after rolling. However, the values of compressive stresses progressively grow in the RD along the strain hardening (see Figure 7a). The differences in residual stresses in RD among the strain rates are negligible beyond the yielding, followed by lower compressive residual stresses for the higher strain rates before the break.

**Figure 8:**
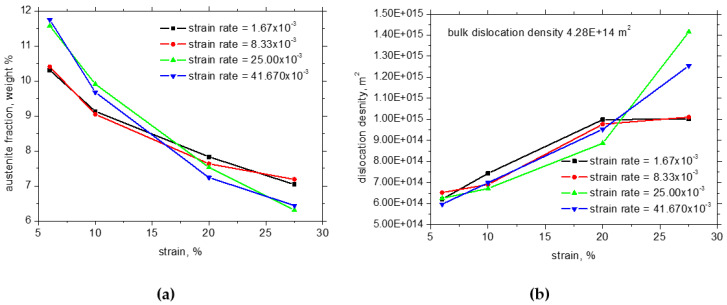
Evolution of austenite fraction and dislocation density in ferrite along *ε*: (**a**) austenite fraction, (**b**) dislocation density in ferrite.

**Figure 9:**
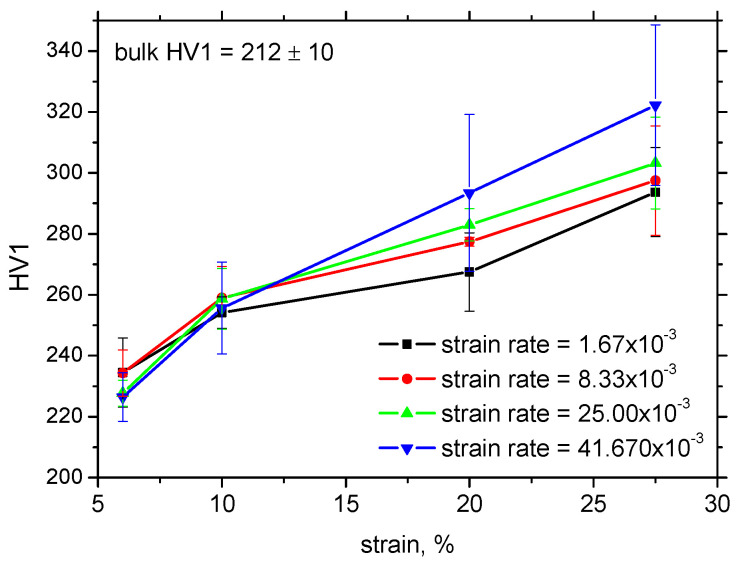
Evolution of *HV1* along *ε*.

The strain hardening mechanism is based on austenite decomposition (phase transformation of austenite to strain-induced martensite—Trip effect) and the superimposing contribution of the interaction of dislocations (their increasing density). Figure 8a demonstrates that the rate of austenite decomposition decelerates at the higher strains [34] and the lower strain rates. On the other hand, austenite decomposition is delayed at the higher strain rates and the lower strains compensated by the lower austenite fraction at the higher strains. Figure 8 shows that the degree of strain-induced transformation of austenite to martensite plays a strong role with respect to *HV1*, since the higher fraction of strain-induced martensite can be linked with the higher dislocation density and the corresponding higher hardness *HV1* at the higher strain and strain rates (also see Figure 9).

It should also be noted that the higher temperatures measured at the higher strain rates should contribute to stabilisation of the austenite phase [4,5]. However, the findings associated with Figure 8 and Figure 9 indicate that this effect is only minor and process dynamics prevails. Furthermore, the higher *β_INT_* at higher strain rates also oppose the higher dislocation density and *HV1* with contrast to *β_INT_* for the lower strain rates. Only a certain release of residual stresses in the RD due to the higher sample heating can be considered when the lower amplitude of compressive stresses for the higher strain rates and strains can be reported, as depicted in Figure 7a.

Finally, it should also be mentioned that the sample breaking is delayed, especially at the lowest strain rate, and the sample breaking is attained earlier for the higher strain rates. For this reason, the true strains and the corresponding density of lattice imperfections are more developed at the higher strain rates and engineering strains. This effect contributes to the higher dislocation density and the corresponding *HV1* value.

### 3.3. Barkhausen Noise Measurements

Two different phases of the MBN in situ evolution can be reported for the RD, TD, and all strain rates. The remarkable descending phase early beyond the yielding is followed by the progressive growth until the breakage. The descending phase can be found in some cases of low-alloyed steels when the Luders region can be found [39,40]. MBN drops down due to the missing interaction among the dislocations (dislocation density is kept nearly unaffected in this region [40]).

**Figure 10:**
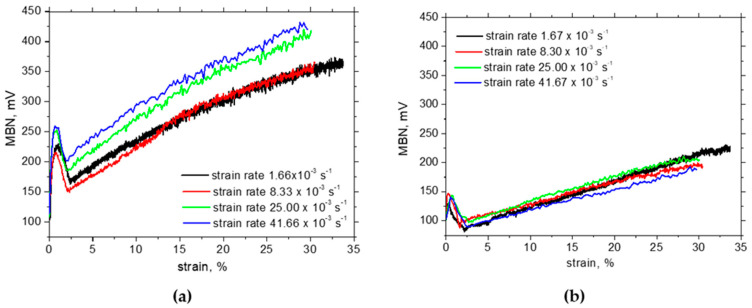
In situ MBN measurements—ViewScan: (**a**) RD, (**b**) TD.

Trip steels do not exhibit this region in the engineering stress–strain curve (see Figure 3; the limited delayed region can be found for the higher strain rates). The in situ MBN evolution beyond the yielding indicates that this phenomenon takes place in the case of Trip steel as well. Moreover, it should be considered that the austenite decomposition is the major mechanism of strain hardening in this phase. The progressive increase in MBN in the RD following the MBN drop is due to the predominating effect of tensile stresses which tends to align DWs along the direction of the stress. Figure 10a also depicts that the rate of MBN growth in the RD decreases since the effect of tensile stress is compensated by the increasing opposition of dislocation density. Furthermore, higher MBNs in the RD for the higher strain rates are due to the higher stresses for the same strains, as shown in Figure 3. It has already been reported that the uniaxial tension tends to align MBN along a direction which is perpendicular to the load when the matrix is yielded [34,39,41]. The moderate growth as depicted in Figure 10 indicates that this effect prevails over the effect of external stresses (tends to align the DWs along the RD) and the effect of growing opposition of the increasing dislocation density. The differences among the in situ MBN in the TD are only minor.

**Figure 11:**
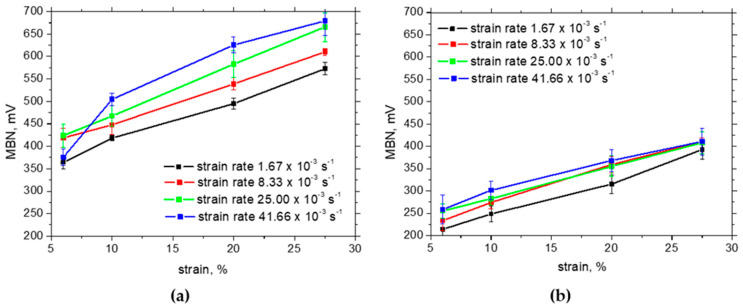
In situ MBN measurements—MicroScan: (**a**) RD, (**b**) TD.

The in situ MBNs measured using MicroScan software are approx. twice higher than those measured using ViewScan software due to the richer MBN signal with respect to the frequency of the obtained MBN signal (compare MBN in Figure 10 and Figure 11). However, the evolutions and the differences among the strain rates and strains are quite similar but only shifted to the higher MBN using MicroScan software.

**Figure 12:**
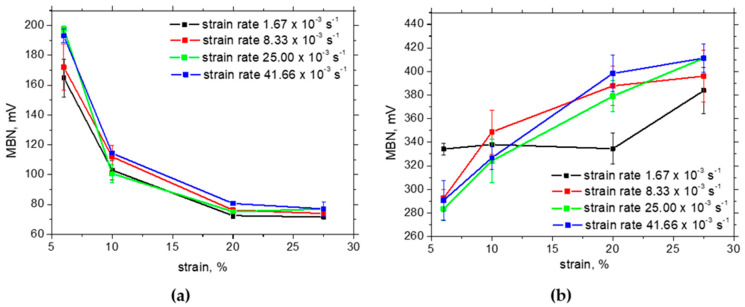
MBN measurements after unloading—MicroScan: (**a**) RD, (**b**) TD.

As soon as the external load is released, MBN evolutions with strain in the RD are remarkably different (see Figure 12). MBN in the RD remarkably decreases due to the increasing opposition of dislocation density and the realignment of DWs along the TD. The evolutions in Figure 12a indicate that the effect of an increasing fraction of the ferromagnetic phase plays no role since MBN drops down despite the paramagnetic austenite being replaced by the ferromagnetic martensite. On the other hand, the MBN measured in situ and post situ in the TD for the higher strain rates is very similar due to the realignment of DWs along the TD [34,39,41]. It should also be mentioned that the initially equiaxed grains become remarkably strained along the direction of tension and the matrix is also gently fragmented [34]. This behaviour remarkably contributes to the developed magnetic anisotropy as mentioned earlier. The realignment of crystals also contributes to the realignment of DWs and explains the increasing MBN in the TD at the expense of the RD [33] (see Figure 12). The influence of ascending temperature especially under the higher strain rates is only minor since the temperature growth is too low with respect to the matrix tempering as well as phase composition.

**Figure 13:**
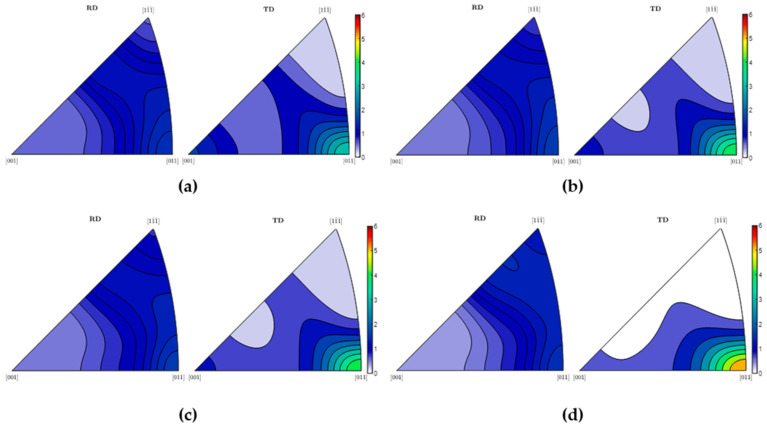
XRD inverse pole figures for strain rate of 8.33×10^−3^ s^−1^: (**a**) bulk, (**b**) 6%, (**c**) 10%, (**d**) 27.5%.

Fe alloys are ferromagnetic bodies of biaxial anisotropy with an easy *[001]* direction of magnetisation in a *bbc* lattice [23,24,25]. Figure 13 shows that after unloading, the easy axis of magnetisation has an inconclusive direction; nevertheless, the TD becomes the easy axis of magnetisation for higher strains. Moreover, Figure 13 clearly proves that the preferential crystallographic orientation of a in the matrix is strongly altered, which in turn can be linked to the realignment of domains and the corresponding DWs. A typical deformation texture was observed for all the measured samples. All typical texture components, typical for rolled steel, were observed: the rotated cubic *{100}<011>*, *{112}<011>*, and *{111}<011>*, which are part of the α_1_-fibre *<110>*‖RD; in addition, the γ-fibre *<111>*‖ND is also very intensive. These two texture fibres prove the TD as the easy axis of magnetisation. The strength of all texture components grows with strain.

**Figure 14:**
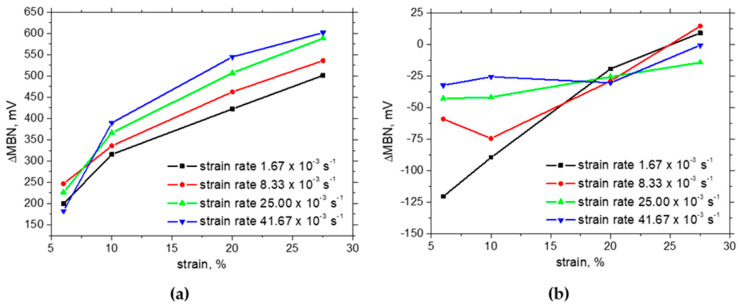
Differences between MBN measured in situ and after unloading—MicroScan: (**a**) RD, (**b**) TD.

The influence of external tensile stresses on MBN can be demonstrated when the MBNs measured in situ and after unloading are subtracted (see Figure 14).

**Figure 15:**
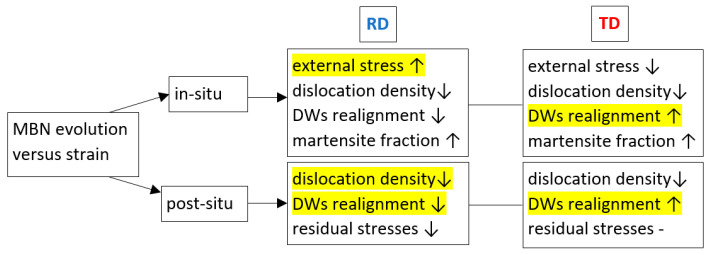
Aspects affecting MBN evolution along with the strain and their predomination (highlighted in yellow).

Increasing external stresses are the main reason for differences between MBN measured in situ and after unloading in the RD. However, this effect is strongly compensated by the alignment of DWs in the TD. Therefore, the differences for the TD are much lower and drop down at the higher strains as contrasted against the RD. For easier navigation with respect to the different aspects affecting MBN evolution (apart from the martensite fraction) and their predomination, please check Figure 15.

**Figure 16:**
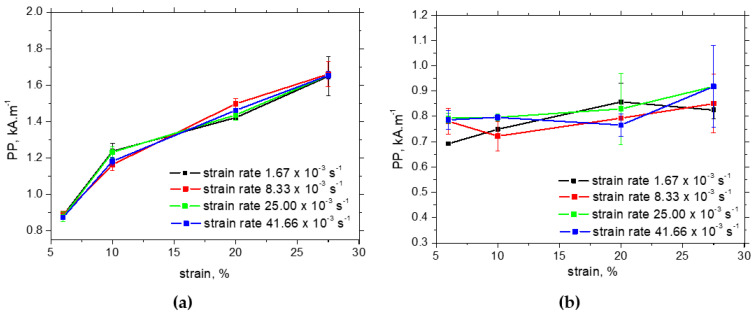
*PP* measurements after unloading—MicroScan: (**a**) RD, (**b**) TD.

The evolutions of *PP* in situ are very flat and quite similar for all strain rates. The measured in situ *PP* values for the RD and TD can be found in the range from 0.85 to 1.05 kA.m^−1^. Also, the evolution of *PP* in the TD after unloading is flat and unaffected by strain rate (see Figure 16b). *PP* values in the TD after unloading are lower by about 0.2 kA.m^−1^ as compared with those measured in situ due to the missing contribution of external stress, which tends to align DWs along the RD. On the other hand, the *PP* values in the RD after unloading exhibit a continuous and remarkable increase (see Figure 16a) due to the increasing opposition of pinning sites (expressed in terms of increasing dislocation density) and the synergistic effect of DWs aligned along the TD [34,39].

**Figure 17:**
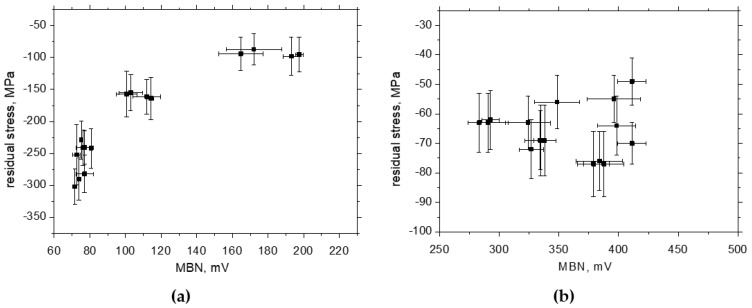
MBN versus residual stresses: (**a**) RD, (**b**) TD.

**Figure 18:**
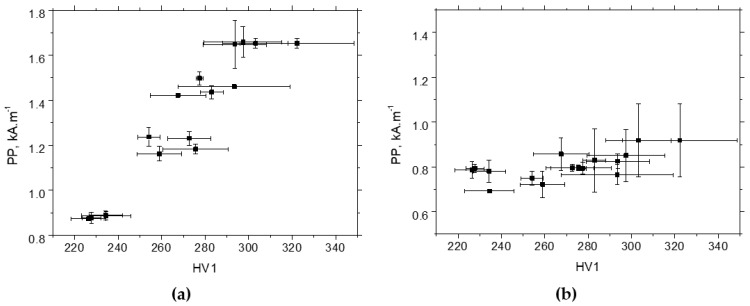
*HV1* versus *PP*: (**a**) RD, (**b**) TD.

One might consider that the increasing magnitude of compressive stresses also contributes to the decreasing MBN in the RD, as depicted in Figure 17 (also see correlation coefficients in Table 3 and Figure 15). However, this effect should be at the expense of decreasing MBN in the TD but is not (see Figure 17) due to the compensation contribution of the realignment of DWs along the TD. Similar conclusions can be reported with respect to the evolution of *PP* versus *HV1*. The correlation between *PP* and dislocation density expressed in terms of *HV1* is very strong (see Figure 18a), whereas the *PP* values grow only a little with *HV1* in the TD (see Figure 18b). Table 1 also proves the close correlation between the dislocation density and *HV1*.

## 4. Conclusions

It can be concluded that the strain rate has no valuable influence on the yield and ultimate strength, whereas the elongation at break drops gently down. The increasing strain rates result in increasing sample heating when the temperature at the low strain rate is about 32 °C near sample breakage as contrasted against 64 °C at the highest strain rate. With almost the same evolution of the plastic work, the Taylor–Quinney coefficient of 0.2 increases with the strain rate up to 0.55. However, the higher dislocation density (1.3 × 10^15^ m^2^) at the higher strains and the strain rates indicates that the correlation between *β_INT_* and the energy consumed by the matrix is low since the effect of delayed sample breakage at the lower strain rate dominates. The residual stresses in the TD are nearly unaffected along the employed strains and strain rates (about −60 MPa), whereas the increasing magnitude of the compressive stresses in the RD can be found especially along with the ascending plastic strains when the residual stress of −100 MPa beyond the yielding is replaced by -300 MPa at the end of the tensile test. Austenite decomposition at the higher strain rates is delayed as contrasted against the lower strain rates, but this evolution is reversed near the sample breakage. The increasing dislocation density strongly correlates with the increasing microhardness *HV1* when the initial *HV1* of 230 beyond the yielding increases up to 300 *HV1*.

In situ MBN in the RD ascends due to the presence of tensile stresses, whereas the realignment of DWs with respect to crystallographic alterations prevails in the TD. The steep increase in MBN in situ in the RD (MBN is growing in the range from 200 mV up to 400 mV) is replaced by the remarkable descending evolution after unloading due to the predominating influence of dislocation density and the superimposing crystallographic realignment of DWs (MBN is dropping down in the range from 180 mV down to 75 mV). On the other hand, MBN in the TD after the unloading is ascending due to the aforementioned realignment of DWs (MBN in TD is growing in the range from 280 mV up to 400 mV). The matrix after plastic straining becomes harder not only from the mechanical but also from the magnetic point of view due to the increased opposition of dislocation density.

The results of this study can be used for many real applications in which Trip steels are employed such as automotive parts that require very high strain hardening during crashes and a large amount of energy absorption. Moreover, information about deformation behaviour under the variable strain rates can also be used in components of high considered formability for the complex reinforcement of systems in order to improve safety in their operation.

## Figures and Tables

**Table 1:** Chemical composition of the employed Trip steel (wt. %).

Fe	C	Mn	Cr	Si	Al	P	Ni	Ti	Cu
Bal.	0.20	1.68	0.06	0.20	1.73	0.02	0.02	0.01	0.03

**Table 2:** Mechanical properties of the employed Trip steel.

Yield Strength	Ultimate Strength	Elongation at Break	Strain Hardening Coefficient	Young’s Modulus
440 MPa	770 MPa	28%	0.29	210 GPa

**Table 3:** Correlation coefficients *ρ_p_*.

		Residual Stress	*HV1*	Dislocation Density
MBN	RD	0.91	−0.85	−0.73
	TD	−0.02	0.9	0.85
*PP*	RD		0.94	0.88
	TD		0.73	0.82

## Data Availability

The original contributions presented in the study are included in the article, further inquiries can be directed to the corresponding author/s.
